# Global Burden of Aflatoxin-Induced Hepatocellular Carcinoma: A Risk Assessment

**DOI:** 10.1289/ehp.0901388

**Published:** 2010-02-19

**Authors:** Yan Liu, Felicia Wu

**Affiliations:** Department of Environmental and Occupational Health, University of Pittsburgh, Pittsburgh, Pennsylvania, USA

**Keywords:** aflatoxin, global disease burden, hepatitis, hepatocellular carcinoma, risk assessment

## Abstract

**Background:**

Hepatocellular carcinoma (HCC), or liver cancer, is the third leading cause of cancer deaths worldwide, with prevalence 16–32 times higher in developing countries than in developed countries. Aflatoxin, a contaminant produced by the fungi *Aspergillus flavus* and *Aspergillus parasiticus* in maize and nuts, is a known human liver carcinogen.

**Objectives:**

We sought to determine the global burden of HCC attributable to aflatoxin exposure.

**Methods:**

We conducted a quantitative cancer risk assessment, for which we collected global data on food-borne aflatoxin levels, consumption of aflatoxin-contaminated foods, and hepatitis B virus (HBV) prevalence. We calculated the cancer potency of aflatoxin for HBV-postive and HBV-negative individuals, as well as the uncertainty in all variables, to estimate the global burden of aflatoxin-related HCC.

**Results:**

Of the 550,000–600,000 new HCC cases worldwide each year, about 25,200–155,000 may be attributable to aflatoxin exposure. Most cases occur in sub-Saharan Africa, Southeast Asia, and China where populations suffer from both high HBV prevalence and largely uncontrolled aflatoxin exposure in food.

**Conclusions:**

Aflatoxin may play a causative role in 4.6–28.2% of all global HCC cases.

Hepatocellular carcinoma (HCC), or liver cancer, is the third leading cause of cancer deaths worldwide [World Health Organization (WHO) 2008], with roughly 550,000–600,000 new HCC cases globally each year (Ferlay et al. 2004). Aflatoxin exposure in food is a significant risk factor for HCC (Wild and Gong 2010). Aflatoxins are primarily produced by the food-borne fungi *Aspergillus flavus* and *Aspergillus parasiticus*, which colonize a variety of food commodities, including maize, oilseeds, spices, groundnuts, and tree nuts in tropical and subtropical regions of the world. Additionally, when animals that are intended for dairy production consume aflatoxin-contaminated feed, a metabolite, aflatoxin M_1_, is excreted in the milk (Strosnider et al. 2006).

Aflatoxins are a group of approximately 20 related fungal metabolites. The four major aflatoxins are known as B_1_, B_2_, G_1_, and G_2_. Aflatoxins B_2_ and G_2_ are the dihydro-derivatives of the parent compounds B_1_ and G_1_ (Pitt and Tomaska 2001). Aflatoxin B_1_ (AFB1) is the most potent (in some species) naturally occurring chemical liver carcinogen known. Naturally occurring mixes of aflatoxins have been classified as a Group 1 human carcinogen by the International Agency for Research on Cancer (IARC) and has demonstrated carcinogenicity in many animal species, including some rodents, nonhuman primates, and fish [International Programme on Chemical Safety (IPCS)/WHO 1998)]. Specific P450 enzymes in the liver metabolize aflatoxin into a reactive oxygen species (aflatoxin-8,9-epoxide), which may then bind to proteins and cause acute toxicity (aflatoxicosis) or to DNA to cause lesions that over time increase the risk of HCC (Groopman et al. 2008).

HCC as a result of chronic aflatoxin exposure has been well documented, presenting most often in persons with chronic hepatitis B virus (HBV) infection (Wild and Gong 2010). The risk of liver cancer in individuals exposed to chronic HBV infection and aflatoxin is up to 30 times greater than the risk in individuals exposed to aflatoxin only (Groopman et al. 2008). These two HCC risk factors—aflatoxin and HBV—are prevalent in poor nations worldwide. Within these nations, there is often a significant urban–rural difference in aflatoxin exposure and HBV prevalence, with both these risk factors typically affecting rural populations more strongly (Plymoth et al. 2009).

Aflatoxin also appears to have a synergistic effect on hepatitis C virus (HCV)-induced liver cancer (Kirk et al. 2006; Kuang et al. 2005; Wild and Montesano 2009), although the quantitative relationship is not as well established as that for aflatoxin and HBV in inducing HCC. Other important causative factors in the development of HCC, in addition to HBV or HCV infection and aflatoxin exposure, are the genetic characteristics of the virus, alcohol consumption, and the age and sex of the infected person (Kirk et al. 2006).

The IPCS/WHO undertook an aflatoxin–HCC risk assessment in 1998 to estimate the impact on population cancer incidence by moving from a hypothetical total aflatoxin standard of 20 ng/g to 10 ng/g (Henry et al. 1999; IPCS/WHO 1998). Assuming that all food containing higher levels of aflatoxin than the standard was discarded and that enough maize and nuts remained to preserve consumption patterns, IPCS/WHO determined that HCC incidence would decrease by about 300 cases per year per billion people, if the stricter aflatoxin standard were followed in nations with HBV prevalence of 25%. However, in nations where HBV prevalence was 1%, the stricter aflatoxin standard would save only two HCC cases per year per billion people. This assessment associated HCC risk with particular doses of aflatoxin; however, these doses do not correspond with actual exposure in different parts of the world, and the two hypothetical values for HBV prevalence, 1% and 25%, were not intended to represent actual HBV prevalence worldwide.

Currently, > 55 billion people worldwide suffer from uncontrolled exposure to aflatoxin (Strosnider et al. 2006). What remains unknown is how many cases of liver cancer can be attributed to this aflatoxin exposure worldwide. Indeed, the Aflatoxin Workgroup (Strosnider et al. 2006), convened by the Centers for Disease Control and Prevention and WHO, identified four issues that warrant immediate attention: quantifying human health impacts and burden of disease due to aflatoxin exposure, compiling an inventory of ongoing intervention strategies, evaluating their efficacy, and disseminating the results.

Addressing this first issue is the aim of our study. We compiled available information on aflatoxin exposure and HBV prevalence from multiple nations in a quantitative cancer risk assessment, to estimate the number of HCC cases attributable to aflatoxin worldwide per year. Shephard (2008) estimated population risk for aflatoxin-induced HCC in select African nations; we expand this to include the rest of the world. We briefly describe interventions that can either reduce aflatoxin directly in food or reduce adverse health effects caused by aflatoxin.

## Materials and Methods

To perform a quantitative cancer risk assessment for aflatoxin-related HCC, we analyzed extensive data sets by nation or world region: food consumption patterns (of maize and peanuts), aflatoxin levels in maize and peanuts, HBV prevalence, and population size. Risk assessment is the process of estimating the magnitude and the probability of a harmful effect to individuals or populations from certain agents or activities. Four steps are involved in estimation of the risk: hazard identification, dose–response analysis, exposure assessment, and risk characterization (National Research Council 1983).

### Hazard identification

Hazard identification is the process of determining whether exposure to an agent can increase the incidence of a particular health condition. Aflatoxin exposure is associated with an increase in incidence of HCC in humans and sensitive animal species (Groopman et al. 2008); in fact, IARC has classified AFB1 naturally occurring mixes of aflatoxins as a Group 1 carcinogen (IARC 2002).

### Dose–response analysis

This second risk assessment step involves characterizing the relationship between the dose of an agent—in this case, aflatoxin—and incidence of HCC. Because of the synergistic impact of aflatoxin and HBV in inducing HCC, the assessment must be done separately for populations with and without chronic HBV infection. Although chronic HCV infection may also have synergistic effects with aflatoxin in inducing HCC, we did not include this effect in the analyses for three reasons: *a*) there is much less overlap worldwide between aflatoxin and HCV exposures in general; *b*) chronic HCV infection usually occurs later in life, whereas chronic HBV infection occurs much earlier, thus the time of overlapped exposure is less significant for aflatoxin and HCV (Groopman J, personal communication); and *c*) much less is known about the quantitative relationship of aflatoxin and HCV in inducing HCC.

For cancer risk assessment, it is traditionally assumed that there is no threshold of exposure to a carcinogen below which there is no observable adverse effect (National Research Council 2008). Cancer potency factors are estimated from the slope of the dose–response relationship, which is assumed to be linear, between doses of the carcinogen and cancer incidence in a population. The IPSC/WHO aflatoxin risk assessment selected two different cancer potency factors for aflatoxin: 0.01 cases/100,000/year/nanogram/kilogram body weight per day aflatoxin exposure for individuals without chronic HBV infection, and 0.30 corresponding cases for individuals with chronic HBV infection. This was based on one cohort study that estimated cancer potency in individuals positive for the HBV surface antigen (HBsAg; a biomarker of chronic HBV infection) and in HBsAg-negative individuals (Yeh et al. 1989), as well as other human studies that assessed cancer potency among either HBsAg-positive or HBsAg-negative individuals. We used these same potency factors for this risk assessment. Because only one of the studies (Yeh et al. 1989) specifically assessed cancer potency in both cohorts, considerable uncertainty may be associated with these potency factors. However, several epidemiological studies confirm that aflatoxin’s cancer potency is about 30 times greater among HBV-positive than among HBV-negative individuals (Kirk et al. 2005, Ok et al. 2007; Qian et al. 1994).

### Exposure assessment

Exposure assessment involves estimating the intensity, frequency, and duration of human exposures to a toxic agent. Specifically, we sought to determine how individuals’ exposure to aflatoxin increases their risk of HCC. Aflatoxin exposure is a function not only of aflatoxin concentrations in maize and nuts but also of how much of these foodstuffs individuals consume in different parts of the world.

Aflatoxin exposure assessment has evolved significantly over the past two decades, largely due to the characterization of biomarkers for both aflatoxin exposure and effect (Groopman et al. 2005, 2008). Before these biomarkers, the primary way to estimate aflatoxin exposure was to observe how much maize and nuts people consumed on average and to measure or assume aflatoxin levels in these foods. By measuring biomarkers such as aflatoxin–albumin adducts in serum or aflatoxin-N7-guanine in urine, it is possible to improve estimations of aflatoxin exposure and how much has been biotransformed to increase cancer risk (Groopman et al. 2008).

Because aflatoxin biomarker data worldwide are limited, we collected data on estimated HBV prevalence in these countries and on maize and nut consumption patterns in different world regions and estimated average aflatoxin exposure or contamination levels in the maize and nuts in different world regions. Where aflatoxin exposure data were not already estimated, we used food consumption patterns and aflatoxin contamination levels to estimate exposure. The studies estimating HBV prevalence were based on HBsAg detection among males and among females in both urban and rural settings across all age groups. Data on maize and peanut consumption in different world regions are adapted from the WHO Global Environment Monitoring System (GEMS)/Food Consumption Cluster Diets database (WHO 2006). We estimated aflatoxin exposure data in different nations from multiple sources through literature searches.

### Risk characterization

This final step of risk assessment integrates dose–response and exposure data to describe the overall nature and magnitude of risk. For our study, this final step consisted of quantifying, across the globe, the burden of aflatoxin-related liver cancer. For each nation, we estimated total number of individuals with or without chronic HBV by multiplying prevalence by population size. To estimate aflatoxin-induced HCC rates within these two populations (with and without chronic HBV infection), we multiplied the corresponding cancer potency factor by aflatoxin exposure estimates. Then we multiplied these values by each nation’s HBV-positive and HBV-negative population sizes to derive total number of aflatoxin-induced HCC cases in each nation. We summed across all world regions to arrive at an estimate for global burden of aflatoxin-induced HCC.

## Results

Table 1 lists the prevalence of chronic HBV infection by world region, as measured by HBsAg in different parts of the world. Although these different estimates involve uncertainty and variability, all data are from literature published in or after 2000, to ensure that the HBV prevalence estimates are as current and as relevant as possible. Countries are grouped by WHO designated regions (WHO 2005): Africa, North America and Latin America, Eastern Mediterranean, Southeast Asia, Western Pacific, and Europe. Some regions were divided into subgroups because of significantly varied aflatoxin exposure and HBV prevalence within the region.

Table 2 provides calculations of maize and peanut consumption in select countries of the world. The GEMS/Food Consumption Cluster Diets database divides countries of the world into 13 groups based on diets. For each group cluster, the GEMS food consumption database has estimated the amount of cereals, nuts, and oilseeds consumed. We thus estimated average maize and nut consumption by individual country. There are limitations to these data because of the clustering into 13 groups (with potentially wide ranges among nations within a group), as well as variability in data quality regarding diet and aflatoxin exposure estimates.

We estimated (based on Tables 1 and 2) or found in the literature the average aflatoxin exposure in different world regions and then calculated the estimated incidence of aflatoxin-induced HCC, with and without the synergistic impact with HBV, in the corresponding populations of each nation and world region (Table 3). Within each WHO-designated region, we found aflatoxin exposures in the most populous nations. The “in general” rows in Table 3 represent a small proportion of each region: nations in which aflatoxin data were not available, or very small nations. For these, we assumed a range for aflatoxin exposure that incorporated the ranges of the nations within the region for which we found aflatoxin data.

These data provide the necessary information to calculate the total estimated cases of aflatoxin-induced HCC cases annually, worldwide. Table 4 lists populations for each relevant nation and world region. Accounting for chronic HBV infection prevalence as shown in Table 1, and the risk estimates for HBV-positive versus HBV-negative individuals in Table 3, the numbers of cases of aflatoxin-induced HCC can be estimated in each world region. These are then summed to produce a global estimate of the number of annual aflatoxin-induced HCC cases. Our estimate is that anywhere from 25,200 to 155,000 annual HCC cases worldwide may be attributable to aflatoxin exposure.

**Figure 1:**
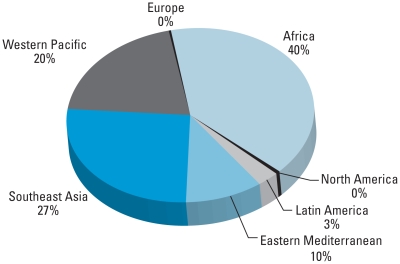
Distribution of HCC cases attributable to aflatoxin in different regions of the world.

Figure 1 illustrates the distribution of HCC cases attributable to aflatoxin globally. The categories denote WHO world regions. Sub-Saharan Africa is the most important region for HCC cases attributable to aflatoxin; Southeast Asia and China (in the Western Pacific region) are also key regions where aflatoxin-related HCC is an important risk. Relatively fewer cases occur in the Americas, Eastern Mediterranean, and Europe. Although Australia and New Zealand are grouped with the Western Pacific region, these nations also have low aflatoxin-induced HCC incidence. It is notable that in Mexico, where HBV prevalence is relatively low but aflatoxin contamination in food is relatively high, aflatoxin appears to be a significant risk factor for HCC among those without HBV (an estimated 152–924 HCC cases per year per 100,000 people).

## Discussion

Aflatoxin contamination in food is a serious global health problem, particularly in developing countries. Although it has been known for several decades that aflatoxin causes liver cancer in humans, the exact burden of aflatoxin-related HCC worldwide was unknown. This study represents a first step in attempting to estimate that burden. We find that at its lower estimate, aflatoxin plays a role in about 4.6% of total annual HCC cases; at its upper estimate, aflatoxin may play a role roughly 28.2% of all HCC cases worldwide. This large range stems from the considerable uncertainty and variability in data on cancer potency factors, HBV prevalence, aflatoxin exposure, and other risk factors in different world regions. The most heavily afflicted parts of the world are sub-Saharan Africa, Southeast Asia, and China.

As indicated in Table 3, populations in developing countries in tropical and subtropical areas are nearly ubiquitously exposed to moderate to high levels of aflatoxin. Aflatoxin is a controllable risk factor in food, yet the parts of the world in which the risk is particularly high have limited resources to implement most aflatoxin control strategies. Much agricultural land in Africa and Asia lies in climatic regions favorable for *A. flavus* and *A. parasiticus* proliferation. Suboptimal field practices and poor drying/storage conditions make crops vulnerable to fungal infection and aflatoxin accumulation. Maize and groundnuts, the two crops most conducive to *Aspergillus* infection, are staples in many African and Asian diets. Because the very poor in these regions cannot afford much food variety, these staples make up a significant portion of their diets, increasing aflatoxin exposure.

Even within the same nation, aflatoxin-induced HCC risk can vary significantly among different populations, hence the large national ranges for risk shown in Table 4. Rural populations generally have higher levels of aflatoxin exposure than do urban dwellers in developing countries (Wild and Hall 2000), because urban populations typically consume more diversified diets than do rural dwellers and may have food that is better controlled for contaminants. In addition, there is a strong seasonal variation in aflatoxin exposure that correlates with food availability (Gnonlonfin et al. 2008; Tajkarimi et al. 2007). Moreover, HBV prevalence is generally higher in rural areas than in urban ones, and higher among males than among females in most places (Plymoth et al. 2009). We present our collected data of HBsAg seroprevalence as a range for countries or populations (Table 1), to account for these variations.

Although many nations that suffer from both high aflatoxin exposures and high HBV prevalence have nominally established maximum allowable aflatoxin standards in food, there is little if any enforcement of these standards in many rural areas. Indeed, the food in subsistence farming and local food markets is rarely formally inspected. Strict aflatoxin standards can even lead to large economic losses for poor food-exporting nations when trading with other nations (Wu 2004). Subsistence farmers and local food traders sometimes have the luxury of discarding obviously moldy maize and groundnuts. But in drought seasons, oftentimes people have no choice but to eat moldy food or starve (Williams 2008).

Multiple public health interventions exist to control the burden of aflatoxin in the body and to prevent HCC. These interventions, described in greater detail in Wu and Khlangwiset (2010), can be grouped into three categories: agricultural, dietary, and clinical. Agricultural interventions can be applied either in the field (preharvest) or in storage and transportation (postharvest) to reduce aflatoxin levels in key crops. They can thus be considered primary interventions. Dietary and clinical interventions can be considered secondary interventions. They cannot reduce actual aflatoxin levels in food, but they can reduce aflatoxin-related illness, either by reducing aflatoxin’s bioavailability in the body or by ameliorating aflatoxin-induced damage. Because aflatoxin-mediated mutations may precede HCC by several years, the effects of reducing aflatoxin exposure on HCC incidence may take time to become apparent (Szymañska et al. 2009).

One highly effective clinical intervention to reduce aflatoxin-related HCC is vaccination against HBV. Vaccinating children against HBV has, over the past 30 years, significantly decreased HBV infection in several regions, including Europe (Bonanni et al. 2003; Williams et al. 1996), Taiwan (Chen et al. 1996), and Thailand (Jutavijittum et al. 2005). This vaccine will, over time, lessen the global carcinogenic impact of aflatoxin, because removing the synergistic impact between HBV and aflatoxin exposure would significantly reduce HCC risk. However, there are currently roughly 360 million chronic HBV carriers worldwide, and HBV vaccination is still not incorporated into many national immunization programs (Wild and Hall 2000). Thus, adopting measures to reduce dietary exposure to aflatoxins is crucial for public health.

Our study highlights the significant role of aflatoxin in contributing to global liver cancer burden. Most cases occur in sub-Saharan Africa, Southeast Asia, and China, where populations suffer from both high HBV prevalence and largely uncontrolled exposure to aflatoxin in the food. Not all risk factors for HCC, including synergistic roles between aflatoxin and other carcinogens, are clearly understood; hence, these estimates for number of global aflatoxin-induced HCC cases have a large range. Although it is impossible to completely eliminate aflatoxin in food worldwide, it is possible to significantly reduce levels and dramatically reduce liver cancer incidence worldwide. The challenge remains to deliver these interventions to places of the world where they are most needed.

## Figures and Tables

**Table 1:** Estimates of HBV prevalence in select countries based on HBsAg seroprevalence.

WHO region/country	References	Chronic HBV prevalence (%)
Africa		
Democratic Republic of Congo	Batina et al. 2007; Mbendi et al. 2001	6–10
Ethiopia	Abebe et al. 2003; Shimelis et al. 2008	6–7
The Gambia	Kirk et al. 2004; Vall et al. 1990	15–20
Kenya	Kiire 1996; Tuei 1994	11–15
Mozambique	Cunha et al. 2007	4.5–10.6
Nigeria	Fasola et al. 2008	13.2
South Africa	Custer et al. 2004; Kiire 1996	3.3–10.4
Tanzania	Hasegawa et al. 2006; Matee et al. 2006; Miller et al. 1998	5–9
Zimbabwe	Kiire 1996; Tswana et al. 1996	10–15
Others	Kiire 1996; Matee et al. 2006; Tayou et al. 2009	9–20

North America and Latin America		
Canada	Minuk and Uhanova 2001; Zhang et al. 2001	1–2
United States	CDC 2009; Cohen et al. 2008; WHO 2005	0.3–2
Argentina	Paraná and Almeida 2005; Torres 1996	0.8–1.1
Brazil	Paraná and Almeida 2005; Torres 1996	2.1–3.4
Mexico	Roman et al. 2009	< 0.3
Others	Paraná and Almeida 2005; Torres 1996	0.5–3

Eastern Mediterranean		
Egypt	Ismail et al. 2009; Lehman and Wilson 2009; Youssef et al. 2009	2.2–10.1
Iran	Kafi-Abad et al. 2009a, 2009b	0.41–0.56
Pakistan	Butt and Amin 2008; Khattak et al. 2002	3.3
Sudan	Elsheikh et al. 2007	6–26
Others	Abou et al. 2009; Batayneh and Bdour 2002; Ameen et al. 2005; WHO 2005	0.65–10

Southeast Asia		
India	Behal et al. 2008; Tandon et al. 1996	2.4– 4.7
Indonesia	Hong et al. 2001; Merican et al. 2000; van Hattum et al. 2003	2.5–5
Thailand	Tanprasert and Somjitta 1993	4.6–8
Others	Alam et al. 2007; Ali et al. 2009; Jafri et al. 2006; WHO 2005	2–7

Western Pacific		
Australia	WHO 2007	< 1
China	Merican et al. 2000; WHO 2007	8–10
Malaysia	Merican et al. 2000; WHO 2007	5
Philippines	Domingo 1997; Lansang 1996; WHO 2007	5–16
Korea	Song et al. 2009; WHO 2007	4–5
Others	Gust 1996; Merican et al. 2000; Nakata et al. 1994	1–10

Europe		
Eastern Europe	Magdzik 2000; Resuli et al. 2009	2–7
Southern Europe	Da Villa 1998; Gogos et al. 2003; WHO 2005	2–7
Western Europe	Hahné et al. 2004; WHO 2005; Jilg et al. 2001	0.5–1

CDC, Centers for Disease Control and Prevention.

**Table 2:** Maize and peanut consumption in select countries.

WHO region/country	Maize^a^ (g/person/day)	Peanut^b^ (g/person/day)
Africa		
Democratic Republic of Congo	57	52
Ethiopia	83	13
The Gambia	57	52
Kenya	248	11
Mozambique	248	11
Nigeria	57	52
South Africa	248	11
Tanzania	248	11
Zimbabwe	248	11

North America and Latin America		
Canada	86	17
United States	86	17
Argentina	86	17
Brazil	63	2
Mexico	300	5

Eastern Mediterranean		
Egypt	136	5
Iran	32	2
Pakistan	35	18
Sudan	57	52

Southeast Asia		
India	35	18
Indonesia	35	18
Thailand	35	18

Western Pacific		
Australia	86	17
China	35	18
Malaysia	35	18
Philippines	59	2
Republic of Korea	59	2

Europe		
Eastern Europe	32	2–10
Southern Europe	148	7
Western Europe	33	10

Data are adapted from GEMS/Food Consumption Cluster Diets database (WHO 2006).

a Including maize, flour and germ.

b Including groundnuts in shell and shelled.

**Table 3:** Estimated HCC incidence attributable to aflatoxin, by WHO region.

WHO region/country	Reference	Aflatoxin exposure (ng/kg body weight/day^a^)	Estimated annual HCC (per 100,000)	
			HBsAg-negative	HBsAg-positive
Africa				
Democratic Republic of Congo	Manjula et al. 2009^b^	0.07–27	0.0007–0.27	0.02–8.10
Ethiopia	Ayalew et al. 2006^b^	1.4–36	0.01–0.36	0.42–10.8
The Gambia	Hall and Wild 1994; Shephard 2008	4–115	0.04–1.15	1.20–34.5
Kenya	Hall and Wild 1994; Shephard 2008	3.5–133	0.04–1.33	1.05–39.9
Mozambique	Hall and Wild 1994	39–180	0.39–1.80	11.7–54.0
Nigeria	Bandyopadhyay et al. 2007; Bankole and Mabekoje 2004^b^	139–227	1.39–2.27	41.7–68.1
South Africa	Hall and Wild 1994; Shephard 2003	0–17	0–0.17	0–5.10
Tanzania	Manjula et al. 2009^b^	0.02–50	0.0002–0.50	0.06–15.0
Zimbabwe	IPCS/WHO 1998	17.5–42.5	0.18–0.43	5.25–12.8
In general^c^	Hall and Wild 1994; Shephard 2008	10–180	0.10–1.80	3.0–54.0

North America				
Canada	Kuiper-Goodman 1995	0.2–0.4^d^	0.002–0.004	0.06–0.12
United States	IPCS/WHO 1998	0.26	0.003	0.08
In general^c^		0.26–1	0.003–0.01	0.08–0.3

Latin America				
Argentina	Etcheverry et al. 1999; Solovey et al. 1999^b^	0–4	0–0.04	0–1.20
Brazil	IARC 2002; Midio et al. 2001; Oliveira et al. 2009; Vargas et al. 2001^b^	0.23–50	0.002–0.50	0.07–15.0
Mexico	García and Heredia 2006; Guzmán-de-Peña and Peña-Cabriales 2005; Torres et al. 1995^b^	14–85	0.14–0.85	4.20–25.5
In general^c^		20–50	0.20–0.50	6.0–15.0

Eastern Mediterranean				
Egypt	Anwar et al. 2008^b^	7–57	0.07–0.57	2.1–17.1
Iran	Hadiani et al. 2009; Mazaheri 2009^b^	5–8.5	0.05–0.09	1.50–2.55
Pakistan	Munir et al. 1989^b^	7–50	0.07–0.50	2.10–15.0
Sudan	Omer et al. 1998	19–186	0.19–1.86	5.70–55.8
In general^c^		10–80	0.10–0.80	3.00–24.0

Southeast Asia				
India	Vasanthi 1998	4–100	0.04–1.00	1.20–30.0
Indonesia	Ali et al. 1998; IARC 2002; Noviandi et al. 2001^b^	9–122	0.09–1.22	2.7–36.6
Thailand	Hall and Wild 1994; Lipigorngoson et al. 2003^b^	53–73	0.53–0.73	15.9–21.9
In general^c^		30–100	0.30–1.00	9.00–30.0

Western Pacific				
Australia	NHMRC 1992; Pitt and Tomaska 2001	0.15–0.18	~0.002	~0.05
China	Li et al. 2001; Qian et al. 1994; Wang and Liu 2007; Wang et al. 2001^b^	17–37	0.17–0.37	5.10–11.1
Malaysia	Ali et al. 1999; IARC 2002^b^	15–140	0.15–1.4	4.5–42
Philippines	Ali et al. 1999; IARC 2002; Sales and Yoshizawa 2005^b^	44–54	0.44–0.54	13.2–16.2
Republic of Korea	Ok et al. 2007; Park et al. 2004	1.2–6	0.01–0.06	0.36–1.80
In general^c^		15–50 (except Australia and New Zealand)	0.15–0.50	4.5–15.0

Europe				
Eastern Europe	Malir et al. 2006^e^	3.5–4	0.04	~1.20
Southern Europe	Battilani et al. 2008; Giray et al. 2007^f^	0–4	0–0.04	0–1.20
Western Europe	IARC 2002	0.3–1.3	0.003–0.01	0.09–0.39
In general^c^		0–4	0–0.04	0–1.2

NHMRC, National Health and Medical Research Council.

a Assuming 60 kg body weight per individual.

b Aflatoxin exposure was estimated by multiplying aflatoxin concentrations in staple foods by consumption rates of those foods (WHO 2006).

c Aflatoxin exposure estimates and consequent HCC cases for all other countries classified in the same WHO region.

d Aflatoxin exposure of 1–2 ng/kg body weight/day was measured in children’s diets; here we assume the adult daily aflatoxin intake is 20% that of children.

e Average daily aflatoxin intake was estimated based on AFB1 contamination levels in Czech maize, multiplied by average daily maize consumption in Eastern Europe. ^f^Average daily aflatoxin intake was estimated based on AFB1 contamination levels in maize of North Italy and Turkey, multiplied by average daily maize consumption in Southern Europe.

**Table 4:** Estimated annual global burden of HCC cases attributable to aflatoxin exposure in HBsAg-positive and HBsAg-negative populations.

		Annual HCC cases	
WHO region/country	Population (millions)^a^	HBsAg-negative	HBsAg-positive
Africa			
Democratic Republic of Congo	68	1–173	1–551
Ethiopia	85	11–288	21–643
The Gambia	1.7	1–17	3–117
Kenya	38	11–450	44–2,270
Mozambique	21	73–361	111–1,200
Nigeria	149	1,800–2,940	8,200–13,400
South Africa	48	0–79	0–255
Tanzania	41	1–195	1–554
Zimbabwe	13	19–50	68–249
Total region	755	2,150–9,300	9,230–50,600

North America			
Canada	33	1	1
United States	300	8	1–5
Total region	333	9	2–5

Latin America			
Argentina	40	0–16	0–5
Brazil	190	4–930	3–969
Mexico	109	152–924	14–83
Total region	562	589–2,980	84–2,060

Eastern Mediterranean			
Egypt	81	51–452	37–1400
Iran	66	33–56	4–9
Pakistan	172	116–832	119–851
Sudan	41	58–717	140–5,950
Total region	569	446–3,720	341–13,200

Southeast Asia			
India	1,150	438–11,200	331–16,200
Indonesia	237	203–2,820	160–4,340
Thailand	63	307–439	461–1,100
Total region	~1,734	1,740–17,300	1,460–27,600

Western Pacific region			
Australia	21	0–1	0–1
China	1,300	1,990–4,430	5,300–14,400
Korea	50	5–29	6–45
Malaysia	28	40–372	63–588
Philippines	90	333–462	594–2,330
Total region	~1,740	2,710–6,510	6,310–21,200

Europe			
Eastern Europe	290	94–114	61–244
Southern Europe	144	0–56	0–121
Western Europe	183	5–24	1–7
Total region	617	99–184	62–372

Total (world)	6,280	7,700–40,000	17,500–115,000
Total annual HCC cases attributable to aflatoxin worldwide		25,200–155,000	

a Data from Central Intelligence Agency 2009.
